# The terminator “Non-sense mediate mRNA decay”: Its role in the termination of intron containing a premature termination codon

**DOI:** 10.12669/pjms.292.3429

**Published:** 2013-04

**Authors:** Younis Skaik

Human genes are composed of exons and interrupted by introns that must be removed by splicing to create a translatable mRNA. Alternative splicing is a vital pathway to regulate gene expression and increase the protein diversity.^1^^,^^2^ In addition to skipping and inclusion of variable exons and usage of alternative splice sites,^3^ intron retention is a third example of alternative splicing, whereby an intron sequence is retained or skipped in the mature mRNA transcript.^4^ Notwithstanding intron retention potentially affects mRNA transport to the cytoplasm^5^ and can insert a premature termination codon (PTC) and hence its degradation by the terminator (Non-sense mediate mRNA decay),^6^ there is an evidence for mRNAs that containing intron and are encoding biologically active proteins.^4^ The markedly increased alternative splicing of genes in human cancers^7^^,^^8^ sheds the light on the possible pathways that might explain the exon containing PCT and whether they are sensitive or resistant to trigger the terminator.^9^ However, to my knowledge, there are no studies that investigated the stealth pathway that the mRNAs transcripts intron-containing PTC^10^^,^^11^ use to escape the radar (terminator). Hence, I would like to address the importance of such studies which will lead to therapeutic options which could suppress the production of the alternative spliced proteins through modulating and strengthen the terminator role.
